# Ewing Sarcoma: An Eponym Window to History

**DOI:** 10.1155/2011/457532

**Published:** 2010-12-01

**Authors:** Timothy P. Cripe

**Affiliations:** ^1^Division of Hematology/Oncology, Cincinnati Children's Hospital Medical Center, Cincinnati, ML7015, OH 45229, USA; ^2^University of Cincinnati College of Medicine, Cincinnati, OH 45267, USA

## Abstract

Ewing sarcoma was named after James R. Ewing, an eminent American pathologist at Cornell who described the first cases in 1921. Although he is best remembered for this singular achievement, Ewing's contributions to the study of cancer were far more profound and influential. He essentially launched oncology as a discipline with the publication of his seminal textbook and founded the major American cancer societies that exist today. His vision of comprehensive cancer centers still drives our research infrastructure. Since his initial report, these organizations have helped us achieve numerous milestones in understanding and treating patients with Ewing sarcoma.

## 1. Introduction

There are thousands of medical eponyms, and keeping track of even a small number is a constant challenge for medical students. At first, such esoteric labels for disease processes or syndromes seem arcane, especially when the underlying molecular defects or mechanisms are known, making much more precise descriptors readily available. But attaching a person's name to a disease or therapy provides a point-of-entry for historical discovery, which may lead to important insights and perspectives that would not otherwise be apparent. The risk of using an eponym, of course, is a reductionist tunnel-vision; one tends to think the person made a singular contribution to medicine. In reality, most historical figures had a far greater impact on their field than a simple eponym would imply. Such is certainly the case with James R. Ewing, who was a pioneer in the field of cancer research, and whose vision continues to steer our cancer research enterprise nearly a century later.

## 2. James Ewing: From Modest Beginnings to Legendary Professor

James Ewing's biography has been recounted in several publications [1–3], but some of the highlights are worth retelling. He was one of five children of a judge, born in Pittsburgh on Christmas Day in 1866. At age 14, he suffered from osteomyelitis of his femur after he was injured while ice skating [2] and was bedridden for months. He occupied much of this time being tutored and entering contests, and in a turn of events that may have influenced his career choice, he won a microscope in one contest for his word play on “Constantinople.” Shortly after completing his medical training, he married Catherine Halsted at the turn of the century, and within two years became a father. Unfortunately, his wife and unborn second son died during childbirth in 1903, and he remained a widower the rest of his life. His resulting personal reclusivity may have contributed to his professional productivity, as his seminal cancer textbook took 10 years to write “including holidays, nights and weekends” [3].

By all accounts, James Ewing was an academic giant. He assembled an impressive *curriculum vitae*, first studying as an undergraduate at Amherst College and then completing his medical training at the prestigious New York College of Physicians and Surgeons in 1891. After a brief stint at the Western Pennsylvania hospital, he did his internship at the Roosevelt Hospital and Sloane Maternity, where he cultivated his interest in anatomic pathology. He volunteered for a year as a contract surgeon to the US army, then in 1899 he managed to land the very first professorship of pathology at the recently minted Medical College of Cornell University in New York City. He published his first textbook only two years later, *Clinical Pathology of Blood: A Treatise on the General Principles and Special Applications of Hematology. *He remained in his position at Cornell for 33 years.

As a young professor, Ewing began to study cancer in animals, such as canine lymphosarcoma, and he quickly became a noted spokesman for cancer research and an avid fundraiser. He established the P. Huntington Fund for Cancer Research in 1902, cofounded the American Association for Cancer Research in 1907, and founded the American Society for the Control of Cancer, now the American Cancer Society, in 1913. In addition, he founded the *Journal for Cancer Research* and teamed with philanthropist James Douglas to create Memorial Hospital of New York, where he later became its first director of research. In his leadership position, Ewing guided the institution's evolution into the nation's first cancer center, now known as Memorial Sloan-Kettering Cancer Center. 

Ewing's most influential academic contribution was his 1919 cancer textbook, *Neoplastic Disease: A Textbook on Tumors*, of which he was the sole author. This comprehensive treatise on cancer spanned early cancer history to modern biologic theory to detailed pathologic descriptions and classifications of all known cancer types. With this publication, Ewing essentially founded oncology as a medical subspecialty. In 1931, Ewing's broad contribution to the cancer field was recognized by *Time *magazine, which featured a sketch of his visage on the cover, calling him “Cancer Man Ewing” [3].

Despite his intensive work schedule, a limp from hip ankylosis, and a nagging facial neuralgia, Ewing managed to maintain his interest in sports, playing tennis on weekends and taking in professional baseball games. Although his adulthood was spent in New York, he remained an ardent fan of the Pittsburgh Pirates and is said to have once skipped one of his own lectures when they were in town to play the New York Giants (then a baseball team). According to accounts, three of his students were also truant and spotted him at the game [3]. Skipping his classes was probably a rare event, as he was said to be “beloved by students and colleagues; a physician of the highest ideals” [3].

Ironically, the Cancer Man died of bladder cancer in 1943 at the age of 76, and at autopsy was also found to have low-grade prostate cancer [1]. His life's impact was evident at his funeral, which was attended by over a thousand people. Ewing shed a bright light on cancer, bringing it into the public eye long before it became a national priority. His vision of establishing six $10 million cancer centers throughout the United States was a blueprint for the current network of National Cancer Institute designated cancer centers, which now number 65 and approach $300 million in core funding. As an Amherst alumnus, James Ewing certainly fulfilled the College's motto, *Terras Irradient*, meaning “Let them give light to the world.”

## 3. Diffuse Endothelioma Tumor

In 1921, two years after he published the first edition of his cancer textbook, Dr. Ewing reported in the Proceedings of the New York Pathological Society several cases of a new bone cancer he called “diffuse endothelioma of bone,” which ultimately became his eponym [4]. In his paper, he described a 14-year-old girl who developed a tumor of the radius that was thought to be an osteosarcoma, which was already well known to clinicians and was usually treated by amputation. Although radiotherapy was increasingly being used for other cancers, osteosarcoma was known to be radioresistant. For reasons not known, this particular patient was given therapies other than amputation, including eight injections of Coley's toxin. That treatment was derived from bacterial erysipelas cultures and used by William Coley via direct intratumoral injection to induce an inflammatory response to the toxin and the tumor [5]. After these injections failed to improve the tumor, she was treated at Memorial Hospital with 12,760 miCu-hr of radium every two weeks for three doses, and surprisingly experienced a complete response by examination and plain films. The effect of radiotherapy suggested at least to some clinicians that the tumor was distinct from osteosarcoma. After the tumor unfortunately recurred, the “conflict of opinion” prompted a biopsy to settle the issue. The pathology was indeed different from osteosarcoma, and Ewing used the vague term “round cell sarcoma.” He thought the cells looked like blood vessels of the bone, and thus termed it “endothelioma of bone.”

In his report, Ewing recounted six other similar cases he had seen in the prior four months. The patients were 14–19 years old, and the primary tumor sites were the tibia, ulna, ischium, skull, and scapula. He described the tumors as slow growing, vascular, and fluctuating in size. On radiographs, he distinguished his series from osteosarcoma: “A large portion or the whole of the shaft is involved, but the ends are generally spared, contrary to the rule with osteogenic sarcoma. The shaft is slightly widened, but the main alteration is a gradual diffuse fading of the bone structure. Bone production has been entirely absent… The radiograph is therefore rather specific.” Based on Ewing's publication, a few years later the noted Boston surgeon, Ernest Codman, referred to this new entity as Ewing sarcoma.

Interestingly, many of the features noted by Ewing in his original report of only a few cases nearly 90 years ago have withstood the test of time. Ewing sarcoma occurs most commonly in adolescents, may appear in flat as well as long bones, most often in the diaphysis rather than the epiphysis, and radiation is a primary treatment modality. Of note, in an ironic crisscrossing of eponyms, Ewing sarcoma is one of the main differential diagnoses of Codman's triangle, the periosteal elevation visible on plain films that often results from a bone tumor.

## 4. Ewing Sarcoma: Historical Milestones

**Figure 1:**
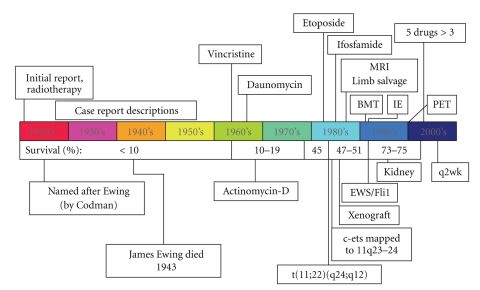
Timeline of historical milestones for Ewing sarcoma. See text for details. Survival data for children diagnosed at age <15 years old are from Rosen et al. [6] and from the Surveillance, Epidemiology and End Results 9 registries as summarized in the work of Smith et al. [7].

In the first 40 years after Ewing's initial description, advances in our understanding of Ewing sarcoma were limited to descriptive isolated case and series reports, which better defined the clinical spectrum of Ewing sarcoma (Figure 1). Surgery and radiation were the only means of therapy until the 1960s, when Ewing sarcoma was among the first solid tumors found to be responsive to chemotherapy including vincristine, daunomycin, and actinomycin D [8–11]. Identification of the activity of ifosfamide and etoposide as single agents each followed over the next two decades [12, 13], though their combination (IE in Figure 1) took another decade to be proven useful [14]. Genetic diagnosis became possible with the identification of a characteristic chromosomal translocation in the 1980s [15, 16], and the mechanism of tumorigenesis began to be elucidated with the cloning of the breakpoint, identifying the EWS-FLI1 fusion early the next decade [17]. These studies also later enabled the consolidation of other tumors, such as the clinically similar but histologically distinct primitive neuroectodermal tumor, into a common tumor family [18]. Preclinical studies were better enabled in the 1980s by the development of a xenograft model [19]. With the increased availability of MRI in the 1980s [20] and FDG-PET in the 1990s [21], new imaging modalities led the way to improved staging, refined surgical approaches including limb salvage [22], and valuable tumor response data [23]. High-dose chemotherapy with stem cell rescue was also pioneered for Ewing sarcoma in the 1990s [24], but after 20 years of use in selected circumstances its utility is still uncertain [25]. Chemotherapy for Ewing sarcoma was further refined in the early 2000s with landmark clinical trials demonstrating that five drugs were better than three [26] and that interval compression (every two-week cycles) was superior to conventional timing (every three-week cycles) [27, 28]. 

As a further illustration of Dr. Ewing's long-lasting impact, the organizations he founded played roles in each these milestones, as some of the work was funded by the American Cancer Society, reported at meetings of the American Association for Cancer Research, and performed at the Memorial-Sloan Kettering Cancer Center and other such centers he envisioned. As we enter the last decade leading toward the century anniversary of his initial case description, further advances are likely to result from targeted molecular approaches that are being intensively studied, such as disrupting the activity of EWS-FLI1 [29] and inhibiting other cell signaling and angiogenic pathways [30].

## 5. Conclusions

While best remembered by his eponym, James Ewing changed the landscape of cancer care and research by single-handedly penning the first comprehensive textbook on cancer and founding what is now the largest charitable organization in the world that supports cancer research, the largest cancer research society in the world, and the largest cancer center in the United States. His vision of comprehensive cancer centers throughout the country, which would bring together diverse experts to study cancer, remains the guiding principle of our nation's cancer research infrastructure. Yet the story of Ewing sarcoma illustrates the slow pace of medical advancement, at least as it occurred in the 20th century. At the time of James Ewing's death, 20 years after he first identified Ewing sarcoma, little progress had been made. It was another 20 years before chemotherapy was used, and another 20+ years before the EWS-FLI1 translocation gave insight into its biology, and yet another 20 years before significant improvements in chemotherapy were realized. Remarkably, his initial description of a new sarcoma has been durable, and the survival of patients today has significantly improved over the past 90 years due to numerous diagnostic, genetic, surgical, radiotherapeutic, and medical advances made possible in part through his organizational efforts. Hopefully such milestones will continue to be realized, perhaps even at a brisker pace, until the day when all patients diagnosed with Ewing sarcoma are curable.
